# Fast micro-computed tomography data of solute transport in porous media with different heterogeneity levels

**DOI:** 10.1038/s41597-021-00803-3

**Published:** 2021-01-20

**Authors:** Stefanie Van Offenwert, Veerle Cnudde, Marijn Boone, Tom Bultreys

**Affiliations:** 1 PProGRess/UGCT, Department of Geology, Ghent University, Ghent, Belgium; 2 Environmental Hydrogeology group, Department of Earth Sciences, Utrecht University, Princetonlaan 8a, 3584 CB Utrecht, The Netherlands; 3 TESCAN XRE, Bollebergen 2B bus 1, 9052 Ghent, Belgium

**Keywords:** Fluid dynamics, Pollution remediation, Imaging techniques

## Abstract

Solute transport processes are influenced by pore-scale heterogeneity. To study this, transient micron-scale solute concentration fields were imaged by fast laboratory-based X-ray micro-computed tomography. We performed tracer injection experiments in three types of porous material with increasing levels of heterogeneity (sintered glass, Bentheimer sandstone and Savonnières limestone). Different Peclet numbers were used during the experiments. For each sample and Peclet number, datasets of 40 to 74 3D images were acquired by continuous scanning with a voxel size of 13.4 to 14.6 µm and a temporal resolution of 15 to 12 seconds. To determine the measurement uncertainty on the obtained concentration fields, we performed calibration experiments under similar circumstances (temporal resolution of 12 seconds and voxel size of 13.0 µm). Here, we provide a systematic description of the data acquisition and processing and make all data, a total of 464 tomograms, publicly available. The combined dataset offers new opportunities to study the influence of pore-scale heterogeneity on solute transport, and to validate pore-scale simulations of this process in increasingly complex samples.

## Abstract (summary)

**Table:** 

Measurement(s)	solute transport in porous media • saline water
Technology Type(s)	micro-computed tomography
Factor Type(s)	Péclet number • Samples with different levels of heterogeneity

Machine-accessible metadata file describing the reported data: 10.6084/m9.figshare.13332644

## Background & Summary

Understanding how dissolved substances are transported through liquids in porous media is a key issue in a wide range of natural and engineering applications, such as remediation of contaminated groundwater^1–3^, waste management^4^, CO_2_ sequestration^5^ and building stone performance^6,7^. Experimental studies have mostly focused on field-scale or cm-scale data^8–10^. However, solute transport is closely linked to solute spreading and mixing, which is impacted by pore-scale heterogeneities^11^. Spreading refers to a change of the solute plume shape without altering its volume^12,13^. Mixing, on the other hand, is associated with increasing the volume occupied by the solute plume by a diffusive smoothing out of concentration gradients^12,13^. Direct quantification of transient, micron-scale solute concentration fields in rock samples is complicated by the high spatial and temporal resolutions that are required^14^. Bultreys *et al*.^15^ and Boone *et al*.^16^ showed that fast laboratory-based micro-computed tomography (micro-CT) can be used to image tracer dispersion experiments at the pore scale in Savonnières limestone. Van Offenwert *et al*.^14^ extended this by quantifying and analysing transient micro-CT concentration fields to investigate solute spreading and mixing in sintered porous glass and in a homogeneous sandstone. The data acquired in these studies covers a range of material heterogeneity (from very homogeneous porous glass to a heterogeneous carbonate rock with multi-scale porosity) at different flow conditions. In this work, we give a systematic overview of the data and how it was acquired, and make all the data freely and publicly available. The combined dataset is uniquely useful to study the influence of pore-scale heterogeneity on mixing and spreading and to validate image-based pore-scale modelling methods, particularly those designed for complex multi-scale porous media^17–20^.

**Fig. 1:**
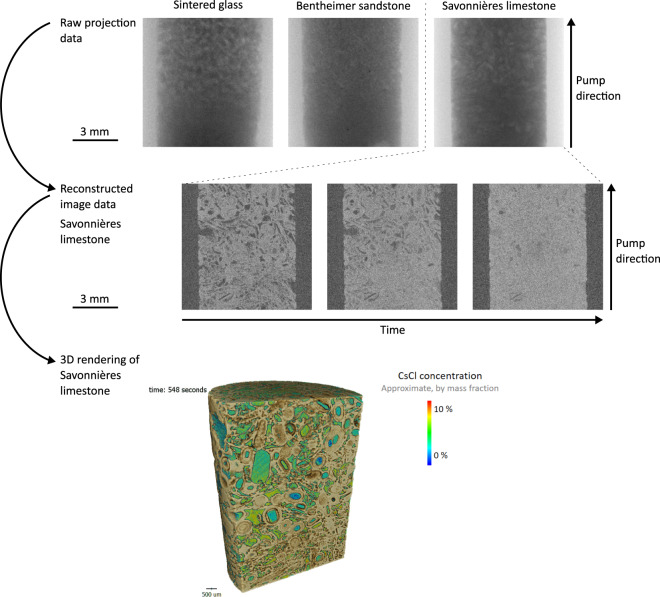
Overview and summary of the different data that was made available on Digital Rocks Portal.

Figure 1 and Table 1 provide an overview of the data made available on Digital Rocks Portal^21–24^. The data of the calibration experiments in a sintered glass sample was used to determine the relation between gray value and tracer concentration^14^. Seven complete 3D time-resolved reconstructed image sequences of the tracer injection experiments in sintered glass, Bentheimer sandstone and Savonnières limestone samples were also made available. For every sequence of the different samples, the matching high-quality images are provided, together with a segmented image of the pore space. An image processing method to calculate tracer concentration fields from this data was described in Van Offenwert *et al*.^14^. In addition, all raw projection data with the metadata are provided in a separate project on Digital Rocks Portal^24^. This allows for reconstructing the data with different reconstruction software and comparing these reconstructed data with the reconstructions we provide^22,23^.

**Table 1:** Overview of different experiments (with the number of 3D images per experiment) in samples with different pore-scale heterogeneity.

Sample	Type of experiment	Number of tomograms
Sintered glass	Calibration experiments: 0, 2, 4, 6, 8, 10 wt% CsCl	8 per experiment (concentration)
	High-quality scan	2 (calibration and tracer injection experiment)
	Tracer injection experiment: 0.25 µl/s	40
	Tracer injection experiment: 0.50 µl/s	49
Bentheimer sandstone	High-quality scan	1
	Tracer injection experiment: 0.25 µl/s	50
	Tracer injection experiment: 0.50 µl/s	50
Savonnières limestone	High-quality scan	1
	Tracer injection experiment: 0.25 µl/s	74
	Tracer injection experiment: 0.50 µl/s	74
	Tracer injection experiment: 1.00 µl/s	71

## Methods

In this section, we provide the details of our sample selection, flow set-up, laboratory-based micro-CT and image processing. The descriptions provide an overview and extension of the ‘Materials and Methods’ section in Van Offenwert *et al*.^14^, the ‘Experimental setup and optimizations for dynamic imaging’ section from Boone *et al*.^16^ and section 3.2.1 of Bultreys *et al*.^15^.

### Sample selection and flow set-up

Two sintered glass samples (L = 20 mm and D = 6 mm; ROBU P0, Germany) were selected for the calibration experiments and for the first tracer injection experiments^14^. Their homogeneous nature and fairly large pores (160 µm–250 µm) facilitated detailed analyses and quantification of the micro-CT images. A Bentheimer sandstone sample (L = 20 mm and D = 6 mm) was selected for the second tracer injection experiment^14^. Its pore size (50–200 µm)^25^ and grain size are smaller compared to the sintered glass sample and in general, Bentheimer sandstone is a bit more heterogeneous^25^. Savonnières limestone (L = 16 mm and D = 6 mm) was used in other dispersion experiments^15,16^. This is an oolitic limestone with ooids and shell fragments which are overgrown by sparite^26^. This rock type is heterogeneous in pore sizes and grain sizes and has large ranges in porosity (22% to 40%) due to local variations^27^.

**Fig. 2:**
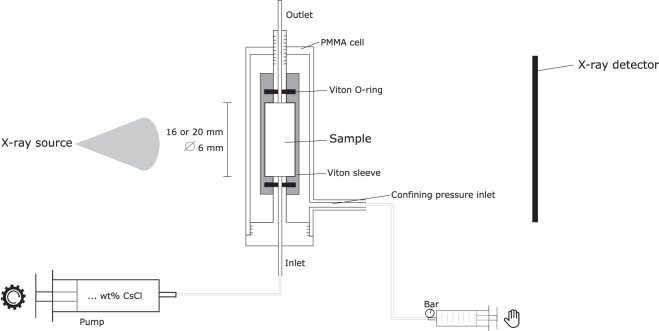
Schematic overview of the experimental set-up to gather the datasets. Adapted from Bultreys *et al*. and Van Offenwert *et al*.^14,15^.

Each sample was placed in a Hassler type polymethylmethacrylate (PMMA) flow cell, which was mounted on a gantry-based micro-CT scanner for *in-situ* scanning^16,28^ (Fig. 2). A Viton sleeve was pressed around the sample by a confining pressure of 0.6 to 1.0 MPa applied with a manual syringe pump. This avoids that the injected tracer solution bypasses the sample during the tracer injection experiments. All elements in the set-up were connected by 1/16” PTFE tubing.

CsCl was selected as tracer because of its high solubility in water and the high X-ray attenuation coefficient of Cs-ions. Due to the high attenuation, higher tracer concentrations lead to higher/lighter gray values in the pore space of the reconstructed images. Because the calibration experiments show that there is a linear relationship between gray value and tracer concentration^14^, the relative concentration can be calculated based on the gray values^29,30^. First, the samples were saturated with demineralized water (after CO_2_ sparging to ensure complete water saturation). For the tracer injection experiments, a solution of demineralized water and 10 wt% CsCl was then pumped through the water saturated sample with a controlled volumetric flow rate using a Harvard PHD Ultra syringe pump or a MilliGat high-precision continuous flow pump (0.25 and 0.50 µl/s for the sintered glass and Bentheimer sandstone sample; 0.25, 0.50 and 1.00 µl/s for the Savonnières limestone sample). The specified flow rates result in different Peclet numbers, which characterize the relative effect of advection and diffusion during solute transport^14,31^. In the calibration experiments, a sintered glass sample was saturated with different concentrations of CsCl (from 0 to 10 wt% CsCl in steps of 2 wt%).

### Laboratory-based micro-CT

A laboratory-based micro-CT system was used for all experiments. Micro-CT is a non-destructive technique to image internal structures and dynamic processes in materials^32–34^. The emitted X-rays are scattered and absorbed by the sample, which causes X-ray attenuation^35^. This is controlled by the X-ray energy and the absorbing material’s density and atomic number. For a mono-energetic beam through a homogeneous material, the Lambert-Beer law describes the attenuation:

$$\frac{{\rm{I}}}{{{\rm{I}}}_{0}}={{\rm{e}}}^{\left(-{\rm{\mu d}}\right)}$$

with I_0_ the initial X-ray intensity, I the intensity of the X-ray photons passing through the object, d the attenuating material’s thickness along the beam (cm) and μ the linear attenuation coefficient of the object (cm^−1^). However, laboratory-based micro-CT systems mainly use polychromatic X-ray beams. This causes image artefacts such as beam hardening in the reconstructed images^32,36^.

Micro-CT works by acquiring radiographs of the sample from different angles, and reconstructing these to a stack of 2D reconstructed slices, which can be rendered into a 3D image. The resulting image consists of voxels, which have a gray value corresponding to the local X-ray attenuation coefficient in the sample. This allows to visualize, and in some cases quantify, differences in density and composition within its internal structure.

The datasets were acquired with a gantry-based micro-CT system designed for *in-situ* imaging, where a X-ray source-detector system rotates around the static sample in a horizontal plane^16,28^. The detector has a thick structured CsI-scintillator. Therefore the micro-CT scanner can be used to scan strongly attenuating objects (like rock material) while still maintaining a high resolution^28^. The X-ray source is a closed 130 kV tube by Hamamatsu with a minimum spot size of 5 µm^28^. The X-ray beam was not filtered at the source or detector due to the concern of losing X-ray flux during fast scanning. However, the sample (surrounded by a Viton sleeve and a thin layer of water) was placed in a PMMA flow cell. This provides some low-energy beam filtering, reducing the impact of beam hardening on the reconstructed gray values. Before the fast scanning experiments, a higher quality pre-scan was taken to determine the static pore structure. The higher quality in the pre-scans is obtained by longer exposure time, a higher frame averaging and a larger number of projections (radiographs) per full rotation and therefore offers a better signal-to-noise ratio^32,35^. During the calibration experiments (sintered glass), the sample was completely saturated with a known tracer (CsCl) concentration. Then, 8 scans were taken continuously (back-to-back) at 12 s per scan. During the tracer injection experiments, scans were gathered continuously at temporal resolutions of 15 s (sintered glass and Bentheimer sandstone sample) or 12 s (Savonnières limestone sample). The linear relationship between gray value and tracer concentration (studied by the calibration experiments), was used to determine the transient micron-scale concentration fields based on the fast images^14^. The detailed scan settings that were used to gather the data are provided in Table 2.

**Table 2:** Detailed scan settings that were used during the different experiments.

Scan settings	Calibration – pre-scan	Calibration – fast scans	Tracer injection – pre-scan (sintered glass and Bentheimer sandstone samples)	Tracer injection – fast scans (sintered glass and Bentheimer sandstone samples)	Tracer injection – pre-scan (Savonnières limestone sample)	Tracer injection – fast scans (Savonnières limestone sample)
Voxel size	6.5 µm	13.0 µm	6.7 µm	13.4 µm	7.3 µm	14.6 µm
Voxel size after resampling and registration	13.0 µm	13.0 µm	13.4 µm	13.4 µm	14.6 µm	14.6 µm
Tube power	6 W	16 W	6 W	16 W	6 W	16 W
Tube voltage	120 kV	130 kV	120 kV	130 kV	100 kV	130 kV
Number of projections per full rotation	2201	600	2201	750	2200	600
Exposure time	120 ms	20 ms	120 ms	20 ms	100 ms	20 ms
Number of averages	5	1	5	1	8	1
Acquisition time	24 min	12 s	22 min	15 s	29 min	12 s
Scans per series	1	8	1	40 to 50	1	71 tot 74

To characterize tracer dispersion, the moment in time at which the tracer plume enters the sample (time T = 0) has to be known. To this end, it is beneficial to include the inlet side of the sample in the imaging. However, the field of view of the micro-CT scanning is limited by the detector size and cannot be extended by scanning the sample at different heights due to the requirement of fast imaging. Therefore, the sample inlet was positioned just inside the detector’s field of view, so that the time T = 0 can be estimated from the acquired radiographs.

### Image processing and analyses

**Fig. 3:**
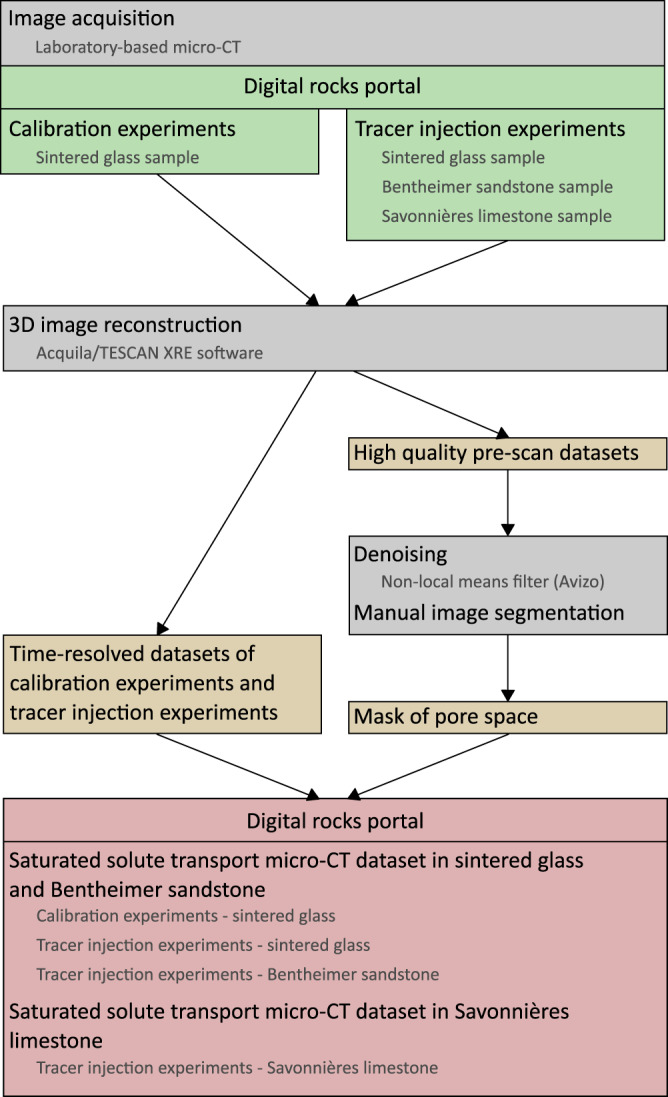
Schematic overview of the workflow from image acquisition to the datasets that are available on Digital Rocks Portal.

The radiographies of every scan are provided on Digital Rocks Portal^24^. Figure 3 provides a schematic overview of the (pre-)processing and analyses steps as well as of the available reconstructed datasets on Digital Rocks Portal^22,23^.

For each scan, the gathered 2D radiographs were first reconstructed to a 3D volume using filtered back-projection using the Feldkamp, Davis and Kress algorithm in Acquila^37^ (TESCAN-XRE, Belgium). More details on this algorithm can be found in Vlassenbroeck *et al*.^37^. After reconstruction, Avizo software was used for image processing and analyses (Avizo 9.5.0, ThermoFisher Scientific). All data was registered and resampled. More specifically, the data of the calibration experiments (sintered glass) were registered and resampled to the higher quality pre-scan. However, for all data from the tracer injection experiments in the three different samples, the higher quality pre-scans were registered and resampled to the different tracer injection datasets (for each sample and each velocity/Peclet number).

For all the tracer injection datasets, the high-quality scans were treated with non-local means filtering and segmented by manual thresholding. This resulted in a mask to identify the pore space from the fast scans of the calibration and tracer injection experiments^15,22,23^. For the Savonnières limestone, the tracer injection datasets are cropped to the overlapping size of the registered and resampled high-quality scan. For this sample, the original high-quality scans are provided besides the segmented masks of the pore space. Furthermore, the macroporosity and microporosity are distinguished (respectively by value 1 and value 2) in these segmented images^23^.

Detailed information about the specific (pore-scale) analyses and interpretations of the calibration experiments and the tracer injection experiments of the sintered glass and Bentheimer sandstone sample can be found in Van Offenwert *et al*.^14^.

## Data Records

For every calibration experiment and tracer injection experiment, we have made the reconstructed images and the (segmented) higher quality images available^22,23^. Detailed information on the available reconstructed datasets is provided in Tables 3–6. The raw micro-CT data are structured correspondingly in a separate project on Digital Rocks Portal^24^.

**Table 3:** Description of the data records of the calibration experiments in a sintered glass sample with the name, number of scans, image size, voxel size and image type (‘Saturated solute transport micro-CT dataset in sintered glass and Bentheimer sandstone’).

Calibration experiments – Sintered glass sample							
Shortened data name	0 wt% CsCl	2 wt% CsCl	4 wt% CsCl	6 wt% CsCl	8 wt% CsCl	10 wt% CsCl	High-quality pre-scan
Number of scans (.raw files)	8	8	8	8	8	8	1
Image size	538x538x380	538x538x380	538x538x380	538x538x380	538x538x380	538x538x380	538x538x380
Voxel size (µm)	12.95	12.95	12.95	12.95	12.95	12.95	12.95
Image type	16-bit gray scale	16-bit gray scale	16-bit gray scale	16-bit gray scale	16-bit gray scale	16-bit gray scale	16-bit gray scale
Data name and type for segmented images of high-quality scans							Segmented high quality pre-scan 8-bit

**Table 4:** Description of the data records of the tracer injection experiments in a sintered glass sample with the name, number of scans, image size, voxel size and image type (‘Saturated solute transport micro-CT dataset in sintered glass and Bentheimer sandstone’).

Tracer injection experiments – Sintered glass sample				
Shortened data name	Volumetric pump velocity 0.25 µl/s	Volumetric pump velocity 0.50 µl/s	High-quality pre-scan (0.25 µl/s)	High-quality pre-scan (0.50 µl/s)
Number of scans (.raw files)	40	49	1	1
Image size	658x658x380	658x658x380	658x658x380	658x658x380
Voxel size (µm)	13.338	13.338	13.338	13.338
Image type	16-bit gray scale	16-bit gray scale	16-bit gray scale	16-bit gray scale
Data name and type for segmented images of high-quality scans			Segmented high quality pre-scan 8-bit	Segmented high quality pre-scan 8-bit

**Table 5:** Description of the data records of the tracer injection experiments in a Bentheimer sandstone sample with the name, number of scans, image size, voxel size and image type (‘Saturated solute transport micro-CT dataset in sintered glass and Bentheimer sandstone’).

Tracer injection experiments – Bentheimer sandstone sample				
Shortened data name	Volumetric pump velocity 0.25 µl/s	Volumetric pump velocity 0.50 µl/s	Segmented high-quality pre-scan (0.25 µl/s)	Segmented high-quality pre-scan (0.50 µl/s)
Number of scans (.raw files)	50	50	1	1
Image size	658x658x380	658x658x380	658x658x380	658x658x380
Voxel size (µm)	13.4	13.4	13.4	13.4
Image type	16-bit gray scale	16-bit gray scale	16-bit gray scale	16-bit gray scale
Data name and type for segmented images of high-quality scans			Segmented high quality pre-scan 8-bit	Segmented high quality pre-scan 8-bit

**Table 6:** Description of the data records of the tracer injection experiments in a Savonnières limestone sample with the name, number of scans, image size, voxel size and image type (‘Saturated solute transport micro-CT dataset in Savonnières limestone’).

Tracer injection experiments – Savonnières limestone sample						
Shortened data name	Volumetric pump velocity 0.25 µl/s	Higher quality pre-scan (0.25 µl/s)	Volumetric pump velocity 0.50 µl/s	Higher quality pre-scan (0.50 µl/s)	Volumetric pump velocity 1.00 µl/s	Higher quality pre-scan (1.00 µl/s)
Number of scans (.raw files)	74	1	74	1	71	1
Image size	658x658x 538	658x658x 538	658x658x 350	658x658x 350	658x658x 500	658x658x 500
Voxel size (µm)	14.6	14.6	14.6	14.6	14.6	14.6
Image type	16-bit gray scale	16-bit gray scale	16-bit gray scale	16-bit gray scale	16-bit gray scale	16-bit gray scale
Data name and type for segmented images of high-quality scans		Porosity mask of higher quality pre-scan (0.25 µl/s) 8-bit		Porosity mask of higher quality pre-scan (0.50 µl/s) 8-bit		Porosity mask of higher quality pre-scan (1.00 µl/s) 8-bit

## Technical Validation

For the reconstructed fast micro-CT scans, the voxelsize is between 13 µm and 15 µm. However, the true image resolution refers to the finest detail distinguishable in the image, which does not only depend on the voxelsize, but also on e.g. image blurring and other artefacts. Although there are no universal measures that fully characterize the true resolution, the Fourier ring correlation has been proposed as a fully automatic quantitative image-based measure without the need for prior information^38^. We calculated this measure using the Fourier Ring Correlation plugin^38–40^ in Fiji (ImageJ) on 15 XY and 15 XZ 2D slices of 3 different fast micro-CT images from the calibration experiments (0 wt% CsCl). This resulted in an average image resolution of 53.98 µm with a standard deviation of 3.17 µm.

The fast micro-CT images suffer from low signal-to-noise ratios. To quantify the effects of this noise, we determined transient pore-scale concentration fields for the provided (reconstructed) datasets^22^. To determine the uncertainty on these concentration fields, we calculated the voxel-based standard deviation of a fixed solute concentration over repeated measurements (calibration experiments in sintered glass sample). This standard deviation was determined to be 27%. By coarse gridding the pore-space (into pore bodies and pore throats), the standard deviation of the solute concentration could be lowered to 1.2%^14^. More details on this, can be found in Van Offenwert *et al*. (2019).

## Usage Notes

The.raw files can be loaded into most image analysis software packages; including for example ImageJ (Fiji). This is open-source software, ideal for the first simple processing steps. Segmented images of the pore space can be used as an input for pore network extraction algorithms like pnextract^41^. Different commercial software like Thermo-Scientific Avizo (or PerGeos), Volume Graphics VG Studio and ORS Dragonfly can be used for in depth analyses and 3D image visualization.
